# Genetic and genomic diversity in the sorghum gene bank collection of Uganda

**DOI:** 10.1186/s12870-022-03770-y

**Published:** 2022-07-29

**Authors:** Subhadra Chakrabarty, Raphael Mufumbo, Steffen Windpassinger, David Jordan, Emma Mace, Rod J. Snowdon, Adrian Hathorn

**Affiliations:** 1 Department of Plant Breeding, Justus Liebig University, Giessen, Germany; 2 Uganda National Gene Bank, National Agricultural Research Laboratories, Kampala, Uganda; 3 Queensland Alliance for Agriculture and Food Innovation, University of Queensland, Warwick, QLD 4370 Australia

**Keywords:** *Sorghum bicolor*, Genetic diversity, Population structure, Cold tolerance, Temperate climate adaptation, Genome-wide association study, Genebank

## Abstract

**Background:**

The Plant Genetic Resources Centre at the Uganda National Gene Bank houses has over 3000 genetically diverse landraces and wild relatives of *Sorghum bicolor* accessions. This genetic diversity resource is untapped, under-utilized, and has not been systematically incorporated into sorghum breeding programs. In this study, we characterized the germplasm collection using whole-genome SNP markers (DArTseq). Discriminant analysis of principal components (DAPC) was implemented to study the racial ancestry of the accessions in comparison to a global sorghum diversity set and characterize the sub-groups present in the Ugandan (UG) germplasm.

**Results:**

Population structure and phylogenetic analysis revealed the presence of five subgroups among the Ugandan accessions. The samples from the highlands of the southwestern region were genetically distinct as compared to the rest of the population. This subset was predominated by the caudatum race and unique in comparison to the other sub-populations. In this study, we detected QTL for juvenile cold tolerance by genome-wide association studies (GWAS) resulting in the identification of 4 markers associated (−log10*p* > 3) to survival under cold stress under both field and climate chamber conditions, located on 3 chromosomes (02, 06, 09). To our best knowledge, the QTL on Sb09 with the strongest association was discovered for the first time.

**Conclusion:**

This study demonstrates how genebank genomics can potentially facilitate effective and efficient usage of valuable, untapped germplasm collections for agronomic trait evaluation and subsequent allele mining. In face of adverse climate change, identification of genomic regions potentially involved in the adaptation of Ugandan sorghum accessions to cooler climatic conditions would be of interest for the expansion of sorghum production into temperate latitudes.

**Supplementary Information:**

The online version contains supplementary material available at 10.1186/s12870-022-03770-y.

## Background

*Sorghum bicolor* [L.] Moench (sorghum) is the fifth most important cereal crop globally and shows remarkable diversity, including five different races, their intermediates, and several crop forms classified as grain, forage, sweet and broomcorn types [1]. Sorghum has extraordinary untapped variation in grain type, plant type, adaptability, productive capacity, and underutilized genetic potential [2]. Because of its wide adaptability to drought and heat, sorghum’s importance is expected to increase with the changing global climate and an ongoing increase in the use of marginal lands for agriculture [3].

In Eastern Africa sorghum is traditionally grown as a food-fodder crop by smallholder farmers in low-input agricultural systems spanning highlands, lowlands, and semi-arid cropping regions. Uganda is located in eastern Africa, south of South Sudan. Archaeological evidence suggests that sorghum’s center of domestication is likely the Ethiopia-Sudan region in the north-east [4]. For ages, sorghum breeders have classified cultivated sorghum into various races (mainly bicolor, guinea, caudatum, kafir, and durra) based on morphological characteristics [5, 6]. Uganda is one of only three countries where all of the five basic races and ten intermediate races of *S. bicolor* are native [7], making it extremely rich in genetic diversity.

The country’s broad sorghum diversity reflects the variety of environments where the crop is grown, mainly on marginal agricultural lands ranging from extremely arid and semi-arid zones in eastern and northern Uganda to cool highlands in south-western regions. The Uganda National Genebank houses a large collection of *S. bicolor* accessions, including a vast range of landraces whose diversity has yet to be capitalized for use in breeding. This germplasm has not yet been fully characterised and evaluated, limiting its utilisation to date in sorghum improvement programs in Uganda and elsewhere. The considerable geographical and topological diversity of Uganda makes this genetic resource a potentially interesting reservoir for genetic analysis and diversity for adaptive traits of interest for sorghum breeding. For example, cool highland areas in the southwestern Uganda are potential sources of diversity for cold-tolerance traits that could help improve sorghum adaptation in temperate cropping regions.

Effective and efficient management of germplasm from a genebank collection is an essential prerequisite for farmers and breeders to identify, extract and exploit the extensive diversity. Genome-wide characterization of untapped genetic resources using genome sequencing technologies provides new opportunities for sustainable breeding and efficient usage of material.

In this present study, the diverse Uganda National Genebank *S. bicolor* collection, representing different agro-ecological zones of the country, was investigated using genome-wide SNP markers and population genetic analysis. The primary objective was to genetically characterize Ugandan (UG) sorghum germplasm in the context of global sorghum diversity, in the absence of morphological data from flowering and maturity for classification into racial groups. As a case study for the value of this resource in adaptive breeding, we furthermore used the available genome data, in association with phenotypic data for juvenile cold tolerance traits, to identify genomic regions enriched with genetic variants associated with low temperature adaptation. The results demonstrate how genebank genomics can help facilitate discovery of economically or biologically important plant diversity and genes as a prerequisite for crop genetic improvement and climatic adaptation.

## Results

### Genetic diversity and population structure analysis

In order to efficiently conserve and utilize the novel UG germplasm, we studied the underlying genetic variation and diversity. The population structure for the UG set was studied using all the 3333 samples and a subset of 12,742 markers from the complete dataset.

**Fig. 1:**
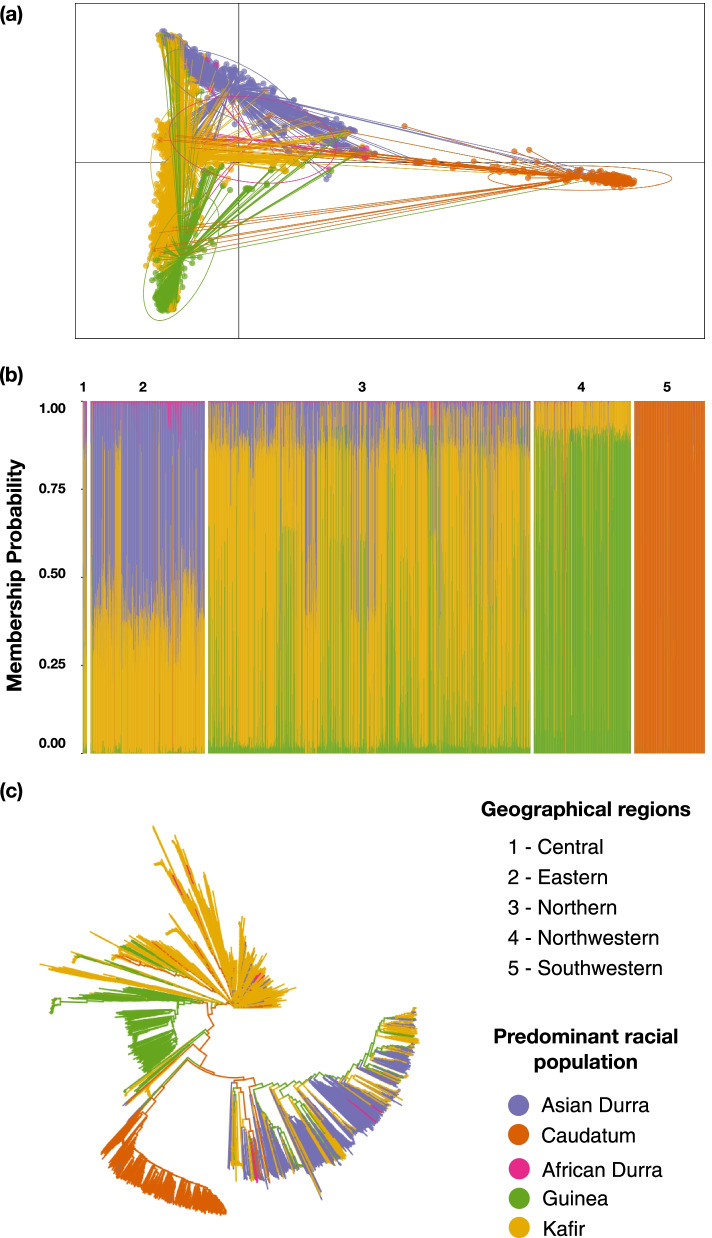
Discriminant analysis of principal components (DAPC) and phylogenetic analysis of 3333 Ugandan accessions. **a** Scatterplot showing the clustering pattern within the population. The axes represent the first two Linear Discriminants (LD) that were retained. Each circle represents an identified cluster whereas each dot represents an accession. **b** Barplot showing assignment of individuals to the five clusters recovered by the DAPC and their racial composition. Each vertical bar represents one individual accession (**c**) Neighbour-joining clustering of the Ugandan germplasm. The numbers are based on the geographical origins and the colours are based on the racial background

Five sub-populations within the germplasm were inferred by the DAPC analysis based on Bayesian Inference Criterion (BIC) (Fig. 1a). Four hundred PCs (27.4% of variance conserved by first three PCs) and four discriminant eigenvalues were retained in the DAPC analysis in order to capture maximum underlying variance. According to the population structure analysis (Fig. 1a, b, Table S1) the accessions from the central, eastern, northern and north western geographical regions did not cluster with any of the described racial groups, whereas the southwestern region showed a distinct genetic pattern with a predominance of caudatum race. The former genetic pattern can be a result of admixed races or it could be a possibility that the samples belonged to the bicolor race, which was not included in the reference global panel because of its dispersed nature. From the results presented in this study we can note that the geographical grouping does not explain the pattern of diversity observed in the germplasm and genetic clustering with the global panel would most likely explain the racial classification better. The distinct pattern of the samples from southwestern region as identified from the DAPC analysis was also confirmed using the phylogenetic tree analysis (Fig. 1c).

**Fig. 2:**
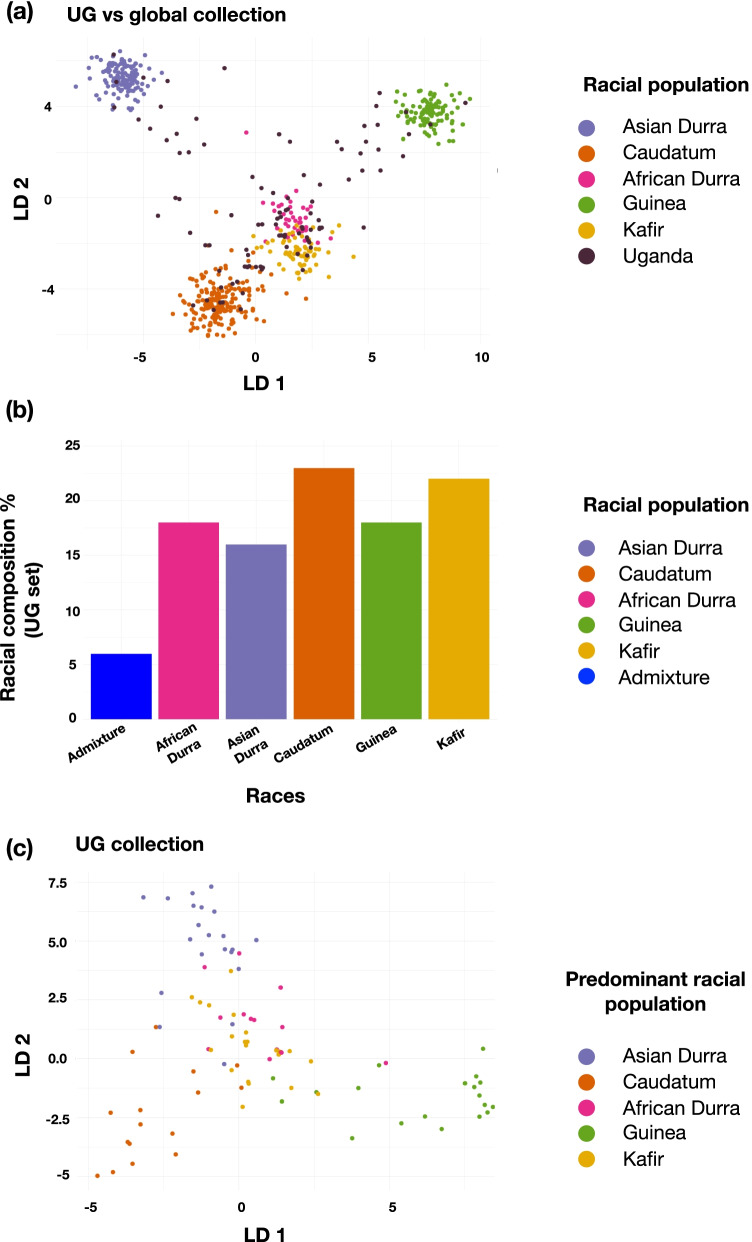
Racial composition and classification of UG samples in comparison to global diversity set. **a** DA loadings (LD) displaying UG clustering (black dots) in comparison to global racial clusters. Each dot represents an individual and the colour code is displayed in the index. **b** composition of the races amongst the UG subset selected for co-analysis with global set. The x axis represents the different races and the y axis indicates the number of individuals that were assigned to the particular race. **c** DA loading of Ugandan representative accessions based on races

With the aim of genetically characterizing the novel Ugandan germplasm and racially comparing it to a well-established global diversity set of geo-referenced sorghum accessions a DAPC co-analysis was performed between the two. As Ugandan accessions outnumbered the global (UQ) set by almost three to one ratio, a random set of UG samples (10 samples from 10 random clusters) were selected for the comparison. All the underlying racial groups from the global collection were identified in the Ugandan germplasm (Fig. 2). Relative to the global set the UG samples were well distributed indicating that majority of the racial diversity was covered by the latter germplasm (Fig. 2a). Based on a threshold of 70%, majority of the samples from this UG subset selected for co-analysis could be assigned to a particular racial group (Fig. 2b) that were used in this study. However, around 5% could not be classified into any single racial category from the global set.

To confirm the racial distribution and diversity in the UG germplasm the DAPC co-analysis with the global set was replicated three times with three random unrelated UG samples. The racial clustering present among the UG set was found to be well dispersed (Fig. 2c, Fig. S1).

Due to significant adaptability differences between the lowland and highland races from the Ugandan diversity set, genetic diversity among the sub groups within the population were tested. Fst between each UG subpopulation from the various geographical regions compared to the entire population revealed genetic variance ranging from − 0.608 to 0.525 (Table S2), indicating presence of significant genetic structuring or genotype variability within each subpopulation within the UG sorghum germplasm.

### Phenotypic variation for juvenile cold stress survival, association mapping and haplotype analysis

A genome-wide association study (GWAS) was conducted for identification of regions of interest associated to juvenile survival under cold stress in two temperate-climate field environments (GG19, GG20) and one controlled-environment climate chamber experiment (CC). Highly significant differences for cold tolerance among genotypes (*p* = 0.000***, Table S3) were found in all experiments. Comparing lowland- and highland genotypes as groups by a one-way ANOVA, highland genotypes showed a superior cold tolerance in all experiments as expected (*p* = 0.015* for GG19, *p* = 0.000*** for GG20, *p* = 0.000*** for CC).

**Fig. 3:**
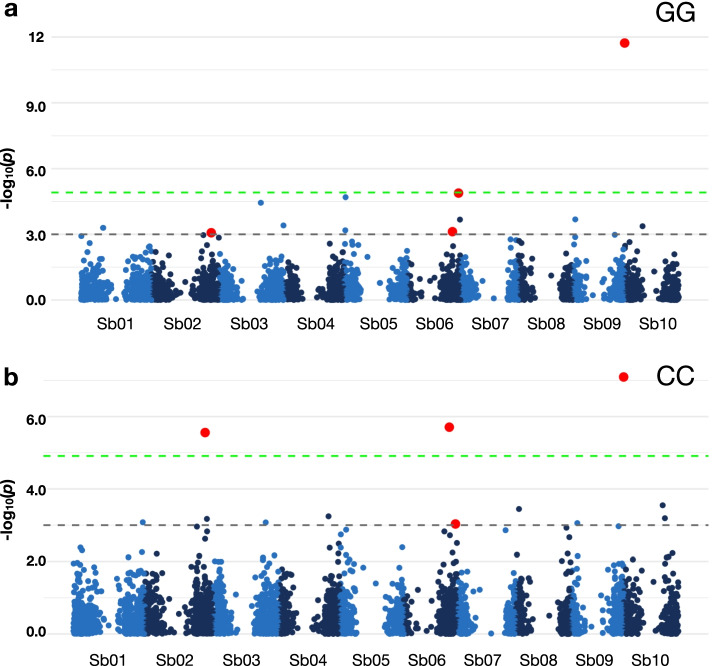
Association mapping of survival under cold stress. The experiments were conducted under (**a**) field condition (Gross Gerau; GG) and (**b**) climate chamber (CC). The grey dotted horizontal line indicates a threshold of genome-wide cut-off at -log10(*p*) > 3.0 while the green line indicates the Bonferroni threshold at -log10(*p*) > 4.9. The selected associated markers which overlapped under both conditions are marked in red

Significant marker-trait associations to juvenile survival under cold stress, consistent across all test environments, were identified on chromosomes Sb02, Sb06, and Sb09 (Fig. 3 a, b, Table S4). We compared these selected genomic regions with previously curated QTL in sorghum QTL-Atlas [8], based on physical position (v3.0) and filtered using category resistance abiotic and subcategory cold tolerance. A sum of nineteen overlapping QTL involved in juvenile cold tolerance were identified (Table S5) in these regions. This result affirms the important role of these genomic regions towards cold adaptation. Furthermore, a novel QTL was discovered on Sb09 which showed the strongest association to juvenile cold tolerance in this UG germplasm.

Comparing protein sequence of orthologous gene segments of sorghum, maize and rice, six genes involved in various cold acclimatization and tolerance were identified (Table S6). For example, the candidate gene associated with the significant peak on chromosome Sb09 is *Sobic.009G260500*, annotated as a tetratricopeptide repeat (TPR)-like superfamily protein coding gene and as a cleavage stimulation factor subunit 3 (Cstf3). This gene family has been shown to have anti-cold response functions in tomato [9] and to be critically involved in heat stress responses in *Arabidopsis thaliana* [10] .

## Discussion

Plant genetic resources like the sorghum collection of the National Genebank of Uganda are extremely important public germplasm resources for local breeders in crop centres of origin and the agricultural and crop research community worldwide. The substantial variation we identified in the Ugandan *S. bicolor* germplasm reflects the highly diverse environments where sorghum grows in Uganda. Accessions belonging to the arid regions of northern and eastern Uganda are well adapted to extreme drought and heat stress, whereas the ones from cold highlands of the southwestern region (Fig. S2) tolerant towards cold temperatures. According to [11] many sorghum landraces exist in this region which has previously not been characterized. To date, there is no available literature demonstrating genetic characterization of sorghum in the southwestern highlands and its role in cold tolerance.

As a result of adaptation to higher altitudes, the accessions from southwestern area are of major interest for breeding programs aiming to expand sorghum production into temperate climates in North America, Asia and Europe, whereas heat and drought stress tolerance are becoming increasingly important for global sorghum production in the face of climate change. The rich genetic resource of the Ugandan genebank sorghum collection has not yet been fully characterised and evaluated, limiting its utilisation to date in sorghum improvement programs in Uganda and elsewhere.

The genetic structure of sorghum has been previously documented in multiple studies using a variety of germplasm collections of different sizes [12]. To our knowledge this is the first report on sorghum genetic diversity using a substantial novel population of 3333 samples from Uganda, a key sorghum centre of origin in comparison to a well characterized global diversity panel [13].

This diversity panel consisted of accessions from the races caudatum, guinea, kafir and two sub populations of race durra, Asian and African. Accessions belonging to race bicolor were not included in this study as they did not form any coherent cluster. The lack of clear differentiation and clustering of *S. bicolor ssp. bicolor* races is not novel and has been previously reported in multiple studies [14–16]. According to literature, durra sorghums reached India before 3000 B.P. From there, they were subsequently introduced around 615 A.D. into Arabic Muslim states [17, 18] and over a period of time developed as distinct groups. Accessions belonging to two subpopulations of durra were included in this study because of their divergent characteristics.

The DAPC method is a good alternative to other population structure analysis software such as STRUCTURE because of its ability to deal with large datasets. Clustering of genotypes presented in this study provide interesting leads for increasing diversity in breeding programs and germplasm utilization. Usually, sorghum is racially classified based on phenotypic and/or morphological data. However, in the absence of appropriate phenotypic information for the collection under the long-day conditions in our field trials, we classified the germplasm solely based on genetic data. Overall, results from DAPC, Kmean and phylogenetic tree analyses were in agreement and provided evidence that the global racial diversity was well-covered in the UG germplasm. However, considerable admixture between racial groups were identified, particularly between genotypes from lowland areas in central, eastern and northern Uganda. This could be caused by movements by local farmers, resulting in infrequent gene flow between these regions, or due to ancestral polymorphisms. According to [19] occurrence of numerous complex genomic interactions involving introgression between the different sorghum races shaped the current genomic diversity and structure within the species. Uganda, being located at the centre of origin and genetic diversity, can be presumed to accommodate accessions possessing patterns of genetic diversity that have been ancestrally inherited. The findings of admixture patterns reported in this study were therefore most likely caused by ancient recombination events and not recent crossovers between the closely related races. However, this phenomenon of non-clustering could also occur if these samples belong to bicolor race, which was not included in the current study. Resolution of this question requires further verification using more phenotypic information from plants grown to maturity under tropical short-day conditions.

Southwestern Uganda comprises predominantly the highland region, which in contrast to the other lowland agricultural areas has a lower average mean temperature [20]. Unlike sorghum accessions from the other geographic regions in Uganda, the accessions from the southwestern region appeared genetically distinct and were comprised predominantly of caudatum race. Because this region includes cool-temperature highland areas, it may contain interesting adaptive diversity for juvenile or reproductive cold tolerance. Population structure and phylogenetic analysis revealed the distinctness of this group compared to the other eco-geographical regions, presumably due to the dissimilarity of the highland cultivation environment and a relatively low exchange of germplasm between high and lowland farmers. Similarly, [21] reported the existence of caudatum and its intermediate races in the highlands of Ethiopia. This confers with the theory that the spread and diversification of crops to different locations can lead to new variants, a process influenced by genotype-by-environment interactions and geographical isolation [22]. According to [23], sorghum accessions from southwestern Uganda tend to have semi-compact elliptic panicles, a well-known characteristic of caudatum and its intermediate races.

Sorghum has a high potential for adaptation to a wide range of environmental conditions. Besides yield and other agronomic traits, the improvement of cold tolerance at juvenile and reproductive stages [24–26] is a major breeding objective for sorghum temperate cropping regions. Early seedling vigour is critical for crop establishment in any environment [27] and vigorous germination and growth under low temperatures is essential for early establishment and weed competition in temperate climates. Improving cold tolerance in the early juvenile stage allows higher yield potential and better maturity. The yield of sorghum is highly temperature dependent, especially between sowing and flowering time [28, 29]. Hence, breeding for juvenile cold tolerance is of utmost importance, especially for temperate European climates. In this study, juvenile survival under low temperature was studied for multi environments. In contrast to emergence and juvenile biomass under cold conditions which have been extensively studied in several publications (e. g. 23,28,29), the trait juvenile survival has received much less attention so far. Though, it is of utmost importance, because a satisfying emergence is worthless if the seedlings later succumb to cold stress. Promising candidates for cold tolerance during juvenile development were identified. Since population stratification was accounted for before performing GWAS, we have reduced the likelihood that genetic background effects are generating spurious associations. We also learned that multiple QTL identified in previous studies [8] and genes known to be involved in cold stress endurance were physically co-located with our QTL, suggesting the importance of our selected genomic regions in this regard. The QTL on Sb09 identified in our study did not overlap with previously reported loci, indicating the importance of this understudied germplam in association to cold tolerance. This draws attention towards the importance of studying novel genetic resources and genome regions containing alleles which can be mined for improvement of cold temperature adaptation.

Given the complex genetics underlying juvenile cold tolerance [29–31], promising approaches like genomic prediction or a genotype-to-phenotype modelling approach can be implemented to assess performance of promising accessions [32]. In addition, after narrowing down genomic region involved in cold tolerance, precise gene editing tools such as CRISPR-Cas9 system can potentially be implemented to validate genes with possible positive effects on abiotic stress tolerance like low temperatures [33]. It would also be interesting to study differential gene expression networks of identified candidate genes in order to to understand their role and elucidate molecular mechanism towards adaptation to abiotic stress responses.

## Conclusions

To our knowledge, this is the first extensive study of the unique and large sorghum germplasm collection conserved at the National Genebank of Uganda. This study focuses on two important aspects, (i) genetic characterization of an underutilized novel germplasm and (ii) dissection of cold tolerance trait within this dataset. The population structure results indicate immense genetic and racial diversity within the germplasm predominated by admixed accessions. Contrast to other geographical regions in Uganda the accessions from the south-western highlands displayed a unique pattern, composed mainly of the caudatum race and genetically isolated from the other subpopulations. This manuscript can be used a base for precisely characterizing novel germplasm based on genetic data. The genomic and phenotypic data collected in this study provide an objective criterion for the selection of accessions for genetic diversity preservation and management, utilization in breeding programs and genetic relationship analysis with other germplasm collections.

A comprehensive investigation involving survival traits, identified multiple key associations and genes underlying the response of juvenile sorghum seedlings to cold stress. The identification of multiple QTL associated to juvenile cold stress reported here can be used by breeders to enhance early-stage chilling tolerance in sorghum. The results provide important new insight for adaptive crops breeding to support the expansion and stability of sorghum production in the face of increasing abiotic stress constraints and climatic change.

## Methods

### Plant materials

A total of 3333 diverse Ugandan *S. bicolor* germplasm accessions (UG set) collected by the Plant Genetic Resources Centre at the Uganda National Genebank were used in this study (Table S1). This germplasm collection represents the entire sorghum diversity from all eco-geographical regions of Uganda ranging from arid and semi-arid areas in eastern parts to the cold highlands of the Kigezi region in southwestern Uganda (Fig. S2). The landraces are well adapted to different local agroecological conditions in terms of elevation, climate, soil and usage. However, these samples are sensitive to photoperiodism and fail to transition from vegetative to reproductive stage if photoperiods are longer than 12 hours, so that morphological classification into racial groups based on floral morphology was not possible in our field trials in Germany. Instead, for racial composition analysis, the collection was compared to a global sorghum germplasm collection of 1033 genotypes (global set) which was previously described by [13].

### DNA extraction and genotyping

Diversity Arrays Technology Pty Ltd. (www.diversityarrays.com) for DNA extraction. The DNA samples were then genotyped using DArTseq, an efficient genotyping-by-sequencing (GBS) platform which enables discovery of genome-wide markers through genome complexity reduction using restriction enzymes. Genotyping was performed fundamentally as described in references [34–36] using the PstI+BanII complexity reduction method. The resulting microarrays were scanned to analyse and score markers by dedicated software DArTsoft (DArT P/L, Canberra, Australia). The sorghum reference genome version v3.1.1 [37] was used for sequence alignment and single nucleotide polymorphism (SNP) calling.

### SNP data filtration

A total of 40,290 SNP markers were reported for the global and UG sets. Firstly, all nonspecific markers and those belonging to supercontigs were removed. The remaining 34,469 were used for further analysis. For racial ancestry analysis, an extremely high stringency was then applied to remove markers and genotypes which exhibited greater than 1% missing data. The global diversity set comprised conversion lines containing introgressed chromosome regions from the Sorghum Conversion Program conducted by Texas Agricultural Experiment Station [38]. Hence, to reduce disparity with UG samples in the co-analysis with the global set, all markers from the specific genomic regions impacted by the conversion program were excluded as follows: Sb06: all markers, Sb07: all markers beyond 40 Mb, Sb09: all markers beyond 46 Mb. Markers with minor allele frequency (MAF) less than 0.01 were also excluded. This set was then imputed using Beagle 5.1 [39] to infer the remaining missing data values. A total of 2331 common markers between the UG and global set were used for the racial ancestry analysis.

### Population structure and genetic diversity study

In order to understand the racial classification and the population structure of the UG sorghum collection, principal component analysis (PCA) and Discriminant Analysis of Principal Components (DAPC) were implemented using the R package Adegenet (2.1.3) [40]. To avoid bias caused by the large size of the UG set, we used a representative subset of the UG set to assign racial groupings in comparison to the global collection. Usually, sorghum racial classification is mainly based on morphological and phenotyping data. However, because the collection is not long-day adapted, the vast majority of the accessions do not reach maturity under the growth conditions in Germany, hence we were unable to classify into racial groups based on morphological data in the field trials performed in this study. However, ongoing analysis of the materials in tropical environments of Uganda will likely provide this missing information in future and enable validation of the genetic classifications.

To select the UG subset representing the meta-population, we initially clustered the entire set into 10 groups followed by randomly sampling 10 accessions from each group. These randomly-selected samples were combined with the global dataset for DAPC co-analysis. To validate the racial assignment of the UG set groups, the DAPC co-analysis was repeated three times, each time using a different set of 10 random genotypes from each of the 10 clusters. The SNP data was converted to the genlight object bit-level genotype coding scheme using the function ‘vcfR2genlight’ of the vcfR tool (https://github.com/knausb/vcfR). After an initial transformation using the PCA analysis, racial composition was subsequently identified for the clusters using discriminant analysis (DA).

To describe the population structure of the UG germplasm and evaluate the racial ancestry of each group in relation to geographical origin of the accessions, the filtered marker set (34,466) was pruned to exclude SNPs which were in strong LD using PLINK software [41]. Pruning was performed using a window of 50 SNPs, step size of 5 makers and r^2^ threshold of 0.5. Finally, a total of 12,742 markers for all UG lines were used to analyse population structure using Discriminant analysis (DA). To elaborate the genetic relationship among the accessions a pairwise distance matrix was established using the tool VCF2Dis (https://github.com/BGI-shenzhen/VCF2Dis), which was then converted to a neighbour-joining phylogenetic tree using the R package ape (5.5) [42] and visualized with R package ggtree (3.0.4) [43]. To study patterns of genetic differentiation, Fst values were calculated for each of the five geographical UG subpopulations against the whole set. This was implemented using the ‘popgen’ function of R package snpReady (0.9.6) [44].

### Phenotyping and association mapping

Juvenile survival under cold stress was evaluated through two field trials at Gross Gerau (GG), Germany (spring 2019 and 2020) and one climate chamber experiment. A UG subset of 444 (field trials) and 255 (climate chamber) accessions representing all agro-ecological zones were used for the association study. For the field experiments (Table S7), all genotypes were sown in micro-plots consisting of single rows (2.5 × 0.7 m) using an alpha lattice block design with two replications. While the recommended sowing time for sorghum in southern Germany is mid-May, the goal of our field experiments was to induce cold stress. Hence, they were sown notably earlier in spring, on April 8 in 2019 and on April 22 in 2020. Even though the mean soil temperatures were relatively high during the course of the experiments (13.8 and 16.2 °C, respectively) and permitted satisfying emergence, occurrence of several cold nights (up to − 1.5 °C) during both the years implied strong stress on the seedlings. Around 4 weeks after emergence, after a period of 7 days from the last occurrence of cold event, the number of surviving plants was scored per plot. For subsequent GWAS analysis, the alpha-lattice adjusted mean value of both years was used. For the climate chamber (CC) experiment, 16 seedlings per genotype were established in 12 × 12 × 12 cm pots. Experiments were designed as randomized complete block design with four replications (Table S8) and the number of surviving plants per pots was scored after 60 days of cold period as a measure for juvenile cold stress tolerance.

After eliminating markers and genotypes with more than 25% missing data points, the dataset was imputed using Beagle 5.1 [39]. It was further corrected for MAF lower than 5%. A total of 4099 markers were used for genome wise association study (GWAS) implemented in R package GenABEL (1.8–0) [45] for juvenile cold tolerance traits. Prior to performing GWAS, population stratification was accounted for by including principal components of the genotypes and genomic kinship matrix [46]. In an effort to reduce the type II error rate and classify a marker–trait association as significant, a threshold of − log10 (*p* value) ≥ 3.0 was defined [47]. Linkage disequilibrium across the entire genome was calculated using the squared allele frequency correlations (*r*^2^) between each pair of SNPs. Haplotype blocks were calculated using an LD threshold of *r*^2^ > 0.7 implemented in the tool LDBlockShow [48], as described by [49].

Candidate genes were selected based on the *Sorghum bicolor* reference genome *v3.1.1* hosted by Phytozome 12 (https://phytozome.jgi.doe.gov/pz/portal.html#!info?alias=Org_Sbicolor). Orthologous genes within selected haploblocks were identified by homology comparisons of the genomic sequences in maize and rice. Protein sequence alignments were conducted by using blastp option of DIAMOND [50].

## Supplementary Information

**Additional file 1: Supplementary Figure 1.** Scatter plot representing DA loading of Ugandan accessions based on races. (a) and (b) are replicate 2 and 3 respectively. Each dot represents an individual and the colour code is displayed in the index.

**Additional file 2: Supplementary Figure 2.** Topographic map of Uganda showing the elevation.

**Additional file 3: Table S1.** DAPC analysis of Ugandan lines based on races and geographical origin. **Table S2.** Population genetics indices for the sorghum accessions from different districts of geographical regions in the Uganda National GeneBank. **Table S3.** Statistical data of the field experiments and the climate chamber experiment. **Table S4.** Genome-wide association study reveals the genetic basis of juvenile cold tolerance trait in sorghum. **Table S4.** Summary of different cold tolerance traits QTL overlapping the haploblock regions. **Table S6.** Summary table of genes identified in the haploblock regions involved in cold stress tolerance. **Table S7.** Conditions of the field experiments conducted in Gross-Gerau, Germany (49°55′ N, 8°29′ E) in 2019 and 2020. **Table S8.** Conditions of the climate chamber experiment.

## Data Availability

The raw data generated and/or analysed during current study has been deposited to the NCBI short-read archive under the Bio-project number PRJNA779225. All variants reported for the Ugandan material as reported by DArTseq are available at 10.5281/zenodo.6535431. Seeds from the collection are deposited in the Uganda National GeneBank in Entebbe and available upon request according to ITPGRFA procedures by contacting the genebank via https://www.pgrc.go.ug/index.php/contactuspgrc.
