# Supplementary material for: Genetic and genomic diversity in the sorghum gene bank collection of Uganda
Source: BMC Plant Biol. 2022 Jul 29;22:378. doi: 10.1186/s12870-022-03770-y (PMC9335971; doi:10.1186/s12870-022-03770-y)
Supplement: Supplementary file 1 — Additional file 1: Supplementary Figure 1. Scatter plot representing DA loading of Ugandan accessions based on races. (a) and (b) are replicate 2 and 3 respectively. Each dot represents an individual and the colour code is displayed in the index. [file 12870_2022_3770_MOESM1_ESM.pdf]

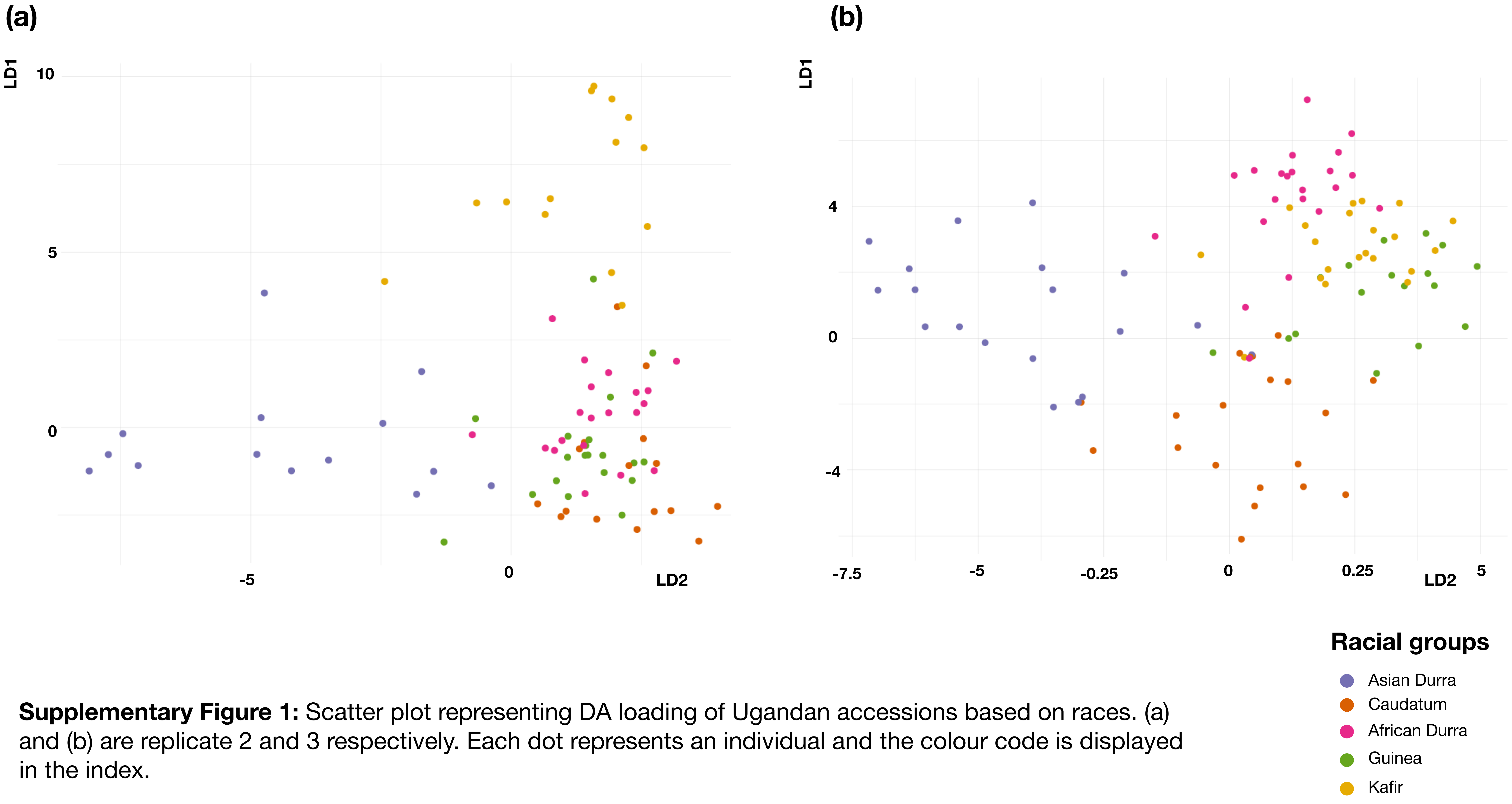

Supplementary Figure 1: Scatter plot representing DA loading of Ugandan accessions based on races. (a) and (b) are replicate 2 and 3 respectively. Each dot represents an individual and the colour code is displayed in the index.
