# Supplementary material for: Genetic and genomic diversity in the sorghum gene bank collection of Uganda
Source: BMC Plant Biol. 2022 Jul 29;22:378. doi: 10.1186/s12870-022-03770-y (PMC9335971; doi:10.1186/s12870-022-03770-y)
Supplement: Supplementary file 2 — Additional file 2: Supplementary Figure 2. Topographic map of Uganda showing the elevation. [file 12870_2022_3770_MOESM2_ESM.pdf]

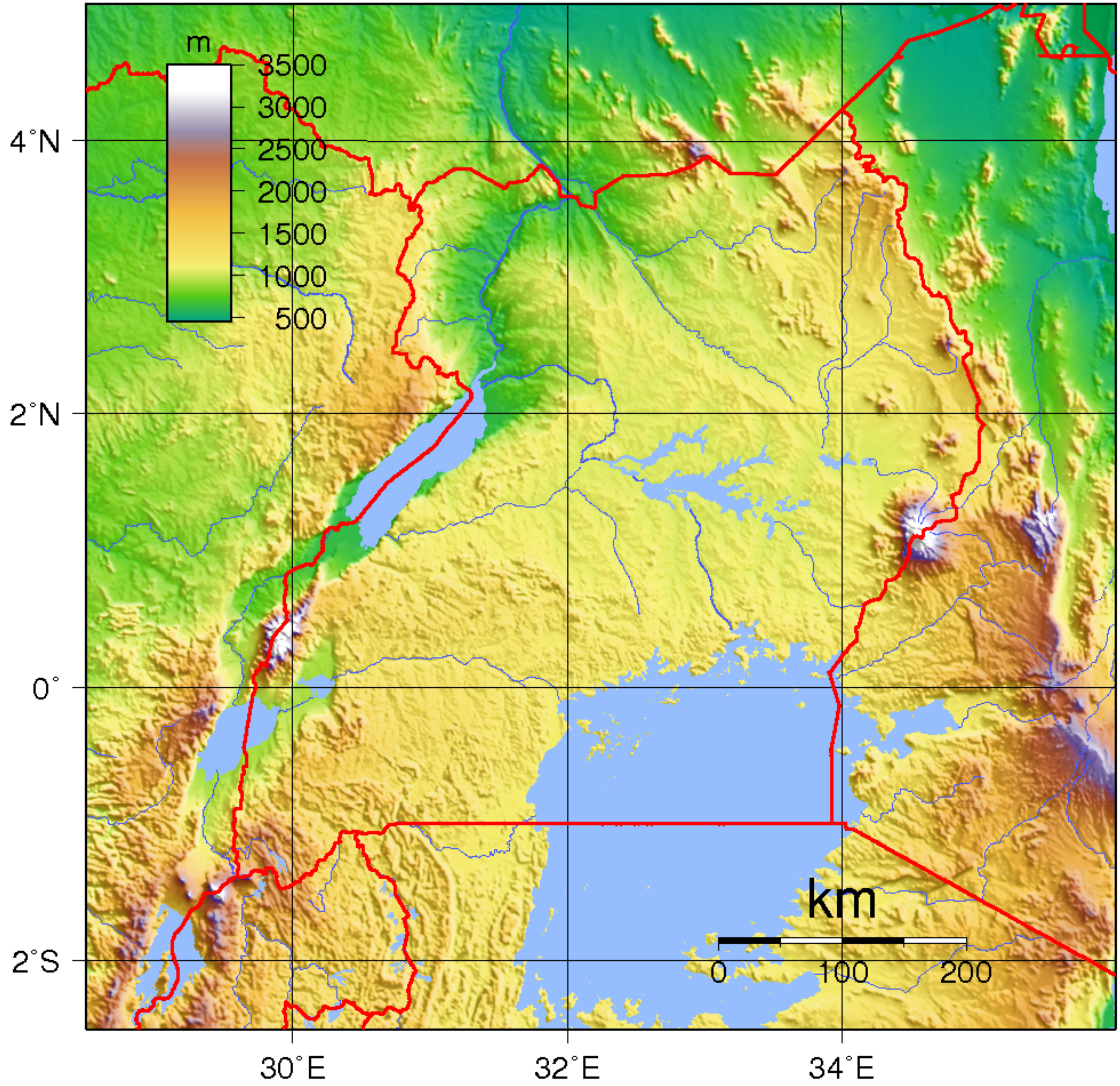

Supplementary Figure 2: Topographic map of Uganda showing the elevation.

Source: https://commons.wikimedia.org/wiki/File:Uganda_Topography.png
